# Seasonality and mycobacterial infectious diseases in animals and humans: is there a generality of seasonal patterns for mycobacterial infections?

**DOI:** 10.1186/s40249-025-01319-3

**Published:** 2025-07-03

**Authors:** Carlos Adrian Vargas Campos, Christine Chevillon, Ahmadou Sylla, Magdalene Dogbe, Kayla M. Fast, Jennifer Pechal, Alex Rakestraw, Matthew E. Scott, Michael W. Sandel, Heather Jordan, M. Eric Benbow, Jean-François Guégan

**Affiliations:** 1 MIVEGEC (UMR Université de Montpellier, CNRS, IRD), Montpellier, France; 2 University of Granada, Granada, Spain; 3 Department of Entomology, Michigan State University, East Lansing, MI USA; 4 Department of Biological Sciences, Mississippi State University, Starkville, MS USA; 5 Department of Wildlife, Fisheries, and Aquaculture, Mississippi State University, Starkville, MS USA; 6 Forest and Wildlife Research Center, Mississippi State University, Starkville, MS USA; 7 Department of Osteopathic Medical Specialties, Michigan State University, East Lansing, MI USA; 8 Ecology, Evolution and Behavior Program, Michigan State University, East Lansing, MI USA; 9 AgBioResearch, Michigan State University, East Lansing, MI USA

**Keywords:** Tuberculous, Non-tuberculous mycobacteria, Human, Animal, Seasonality, Temporal dynamics, Disease transmission

## Abstract

**Background:**

Seasonal patterns of mycobacterial infections affecting humans and animals remain a complex and understudied aspect of infectious disease dynamics. These intra-annual patterns are increasingly relevant in the context of global climate change, which may influence the timing and geographic spread of these diseases. A better understanding of such patterns could improve surveillance, prevention, and control strategies.

**Methods:**

We conducted a mixed-methods bibliometric review combining bibliographic searches and scoping analysis to synthesize decades of research on the seasonality of mycobacterial infections in humans and animals. We systematically searched three major scientific databases—Scopus, PubMed-MEDLINE, and Web of Science—for articles published between 1971 and April 2023. From an initial dataset of 1830 unique articles, we identified and analysed 122 studies that met predefined inclusion criteria. We extracted information on pathogen type, statistical methods, geographic location, and host species. In addition, we conducted a co-citation network analysis to identify key methodological influences and research clusters.

**Results:**

The retained studies encompassed tuberculosis, Buruli ulcer, bovine tuberculosis, and other mycobacterial diseases such as leprosy and Johne’s disease. Most articles focused on tuberculosis in humans, followed by Buruli ulcer caused by *Mycobacterium ulcerans*. There was a marked increase in studies on seasonal trends in tuberculosis and Buruli ulcer over time, with notable variation in geographic and methodological coverage. Research was heavily concentrated in the northern hemisphere, especially in China, while southern regions remained underrepresented. Advanced statistical tools, including generalized linear models and time-series analyses, were instrumental in detecting seasonality, particularly for tuberculosis and Buruli ulcer.

**Conclusion:**

Seasonality appears to be a common yet understudied feature of many mycobacterial infections. Greater interdisciplinary collaboration and the use of appropriate analytical tools are essential to better understand these patterns, especially in underrepresented regions. Addressing methodological and geographic gaps will be crucial to improve responses to these diseases in a changing global environment.

**Supplementary Information:**

The online version contains supplementary material available at 10.1186/s40249-025-01319-3.

## Background

Seasonal patterns of infectious diseases have intrigued both researchers and medical doctors since at least in the Greek Hippocratic period of the V^th^ century BC. Seasonality in infectious diseases refers to recurrent, periodic fluctuations in incidence that occur at regular intervals throughout the year, often driven by climatic, ecological, or sociobehavioral factors [1]. Distinct peaks in diseases such as varicella zoster virus during spring and seasonal influenza viruses during winter underscore the significant influence of seasons on epidemic patterns, resulting in temporal waves of cases throughout the year [2]. Mycobacterial pathogens can be broadly classified based on their primary host. Human pathogens include *Mycobacterium tuberculosis* (TB, the causative agent of human tuberculosis) and *Mycobacterium ulcerans* (MU), responsible for Buruli ulcer (BU). Animal-specific pathogens comprise *Mycobacterium avium paratuberculosis* (MAP), which causes Johne’s disease, and *Mycobacterium bovis* (bTB), the agent of bovine tuberculosis. Notably, bTB represents a shared pathogen capable of infecting both animals and humans, illustrating the zoonotic potential of certain mycobacterial species.

While TB, bTB, and MAP have been the focus of many studies, the seasonal dynamics of other mycobacterial disease cases and their causative agents remain largely unexplored, with minimal research compared to other major infectious diseases [1].

TB research, with over 36,840 publications from 1904 to April 2023 in Web of Science Clarivate Analytics™ scientific database, serves as a key model for studying mycobacterial diseases. Despite advancements, in 2021, an estimated 10.6 million people developed TB globally [3], with the zoonotic variant bTB contributing up to 12% of human TB-like cases in certain regions [4]. MAP infections, namely, Johne’s disease, exhibit geographic variation, being most prevalent in eastern Asia and less common in northern America and Europe [5]. The economic burden of Johne’s disease and bovine tuberculosis on animal industry is substantial, with losses reaching dozens of millions of dollars or euros annually, underscoring the need for improved disease management, including potential insights from understanding seasonal trends [6, 7].

This mixed-methods review evaluates global research on the Mycobacterium tuberculosis complex (MTBC) and non-tuberculous mycobacteria (NTM) infection seasonality. We highlight pronounced seasonality in TB, BU and its etiological agent MU, and bTB. Additionally, we underscore the importance of integrating studies on mycobacterial pathogens by using an ecosystem-based approach, that is, associating both pathogen population dynamics in the environment and host disease case dynamics, to gain a comprehensive understanding of disease ecology, which is vital for improving veterinary and human health strategies [8]. In addition, a co-citation network analysis revealed key methodologies and conceptual frameworks driving research on mycobacterial seasonality. We identified time-series analysis as a pivotal technique for uncovering seasonal patterns and understanding pathogen dynamics often overlooked in traditional studies. By demonstrating the value of these approaches, this review highlights opportunities for advancing future research in animal and human infectious disease dynamics in the context of major global changes. The aim of this review is to determine whether a general pattern of seasonality exists across mycobacterial infections in humans and animals, and to assess how different methodological and geographic approaches influence the detection of these temporal trends.

## Methods

### Search strategy and selection criteria

This study utilized a mixed-methods approach, combining bibliometric analysis with a scoping review, to explore global research on mycobacterial disease seasonality. The analysis included searches across three major databases: Scopus™, PubMed-MEDLINE™, and Web of Science Clarivate Analytics™, from January 1971 to 7 April 2023. The search strategy employed a comprehensive set of terms related to mycobacteria and seasonality, using Boolean operators (including, “AND”, “OR”) and truncations to ensure broad coverage (see Supplementary material). Articles were organized using Mendeley Desktop™ (version 1.19.8) citation software, which also facilitated the removal of duplicates.

C.A.V.C., J.-F.G. and C.C. initially screened papers by analysing titles and abstracts. They considered inclusion criteria: (1) peer-reviewed papers in indexed scientific journals; (2) papers with concise information on seasonality using mathematical or statistical tools based on primary data; (3) empirical research; (4) studies published in English, Spanish, and French. Exclusion criteria were: (1) papers lacking original empirical data on mycobacterial seasonality, and (2) reviews without original research.

### Bibliographic collection and analytical strategies

The selected articles were analysed for bibliometric, geographic, and taxonomic information, focusing on mycobacterial species and their resulting infections in humans, animals, or both. Key details such as publication year, author names, journal names, author count per paper, and institutional affiliations and countries were extracted. Additionally, seasonal data and statistical or mathematical methods used in each study were recorded. To explore the intellectual landscape of mycobacterial seasonality research, co-citation networks were then constructed. These directed graphs highlighted connections between documents through shared citations, enabling the identification of influential works, prominent research clusters, and emerging trends [9]. PageRank© gauged article relevance, prioritizing highly cited works and those cited by influential publications [10]. PageRank© is a link analysis algorithm, with the purpose of estimating the relative importance of these scientific documents, and taking into consideration authority hubs and influencing links. This method surpasses total citation count for assessing paper significance [11].

### Quality assessment and extraction of seasonal trends

The evaluation of seasonality patterns encompassed reading paper contents and analysing results and graphics within publications. We categorized the different methods used for exploring seasonal trends into four different classes. This classification was adapted from Christiansen et al. (2012), who summarized the main approaches employed in seasonality studies in infectious disease epidemiology [12]. Studies were classified into four categories: “Class zero” for no precise statistical method and often pretextual evidence with no formal demonstration of the existence of seasonality; “Class one” included descriptive statistics of such pattern (such as, mean, median, standard deviation, interquartile range, prevalence, incidence); “Class two” for those based on inferential methods (including, *χ*^2^ test, analysis of variance, Pearson’s correlation test); and “Class three” that included papers with disease time-series evaluation methods (such as, generalized linear models, autoregressive integrated moving average, autocorrelation functions, LOWESS (Locally Weighted Scatterplot Smoothing), wavelet analysis or more sophisticated approaches). A representative example for each class is included in Supplementary Materials.

### Integration of bibliometric and scoping review analyses

This review employed a mixed-methods design integrating bibliometric analysis and scoping review. The bibliometric component described publication trends, geographic origins, and citation networks specific to seasonality in mycobacterial research. The scoping review complemented this by synthesizing detailed evidence on seasonal patterns and analytical methods.

### Gap analysis

**Fig. 1:**
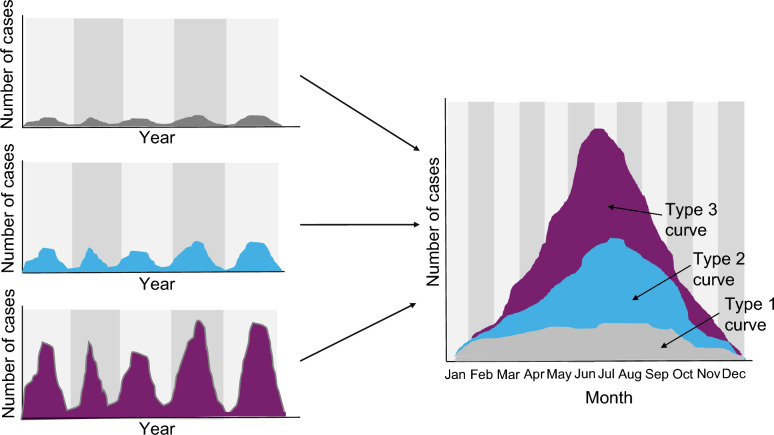
Representation of seasonal cyclicity in mycobacterial infections

For articles classified as one through three, ranging from preliminary evidence to formal demonstrations of mycobacterial seasonality, a secondary classification system was applied to evaluate the robustness and depth of their findings. This classification examined the types of results presented and their methodological rigor. Details of the scoring methods and statistical techniques used are provided in Panel 1 and Fig. 1. Analytical tools such as *R*Studio™ (version 2023.06.1 + 524) with the ggplot2 and bibliometrix [13, 14], Systat 13™ (version 13.2, Systat Software, Inc., San Jose, USA), and Stata™ (version 15, StataCorp LLC, Texas, USA) were employed for data representation, analysis, and visualization.

Panel 1. Seasonality of mycobacterial infectious diseases Seasonality, or seasonal cyclicity, is a characteristic of time-series data observed across diverse fields, including physical oceanography, animal population dynamics, and economics. It reflects regular annual changes in measured values, such as sea surface temperature, population size, or commodity prices, which repeat predictably across years. In infectious disease ecology, seasonality corresponds to periodic, intra-annual surges in disease incidence associated with seasonal transitions, such as summer versus winter or dry versus rainy periods. This phenomenon is a hallmark of many acute infectious diseases, playing a critical role in their dynamics and public or animal health importance [1]. Despite its recognition, the mechanisms underlying seasonal epidemic waves remain poorly understood, and long-term datasets for animal diseases are most often lacking. Seasonal variation in infectious disease occurrence may range from subtle and difficult to detect to pronounced, producing sharp epidemic peaks. To systematically evaluate seasonal patterns in animal and human mycobacterial infections, this review categorized epidemics or epizooties into three types based on their intensity. Type one seasonal epidemics represent slight increases in case numbers or mycobacteria abundance that do not lead to marked outbreaks, receiving a score of one. Type two epidemics, characterized by moderate seasonal increases following a flat, bell-shaped curve, were assigned a score of two. Finally, type three seasonal epidemics demonstrate rapid and pronounced peaks in cases or abundance, earning a score of three (see Fig. 1). This classification framework was applied to assess the role of statistical and mathematical methods in revealing seasonal dynamics. Two referees, the first and last authors of this review, independently analysed the seasonal shapes observed in the included studies, assigning scores according to the defined criteria. This double-blind scoring process was validated using Cohen’s Kappa statistical test, ensuring reliability. Average seasonal scores were then compared across the statistical and mathematical categories used in the studies (see Table 1). Our findings underscore the hypothesis that with the adoption of advanced statistical and mathematical techniques from fields beyond human and veterinary medicine, seasonal trends in mycobacterial infections can be revealed even when they are not immediately apparent through simple visual inspection

## Results

### Characterizing the scientific literature

**Fig. 2:**
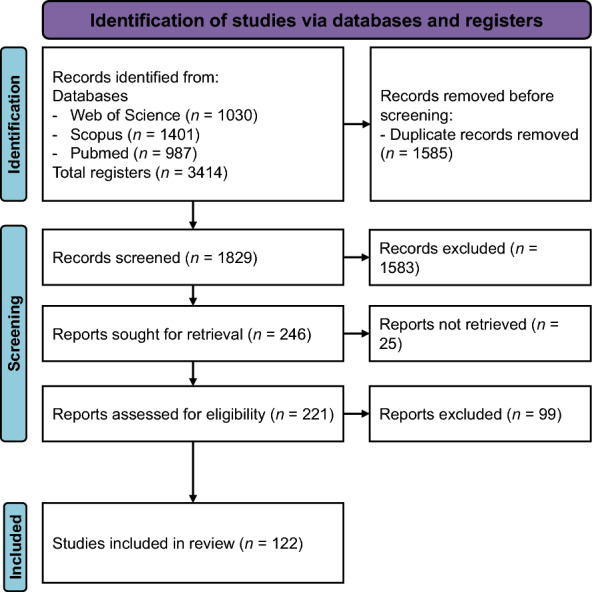
PRISMA flowchart

In this scientific review, we initially identified 3414 publications from three bibliometric databases (Scopus™, PubMed-MEDLINE™, and Web of Science™) covering the period from 1 January 1971 to 7 April 2023. After removing duplicates, the corpus was reduced to 1830 unique articles. During the screening process, 1609 articles were excluded because they lacked relevant or sufficient information on seasonality (Class zero). Of the remaining 246 papers, we identified 24 articles that likely met our inclusion criteria but for which the full texts could not be obtained through institutional access or open sources and 1 article not accessible due to errors in publication records. These studies were excluded due to lack of access, which precluded methodological assessment. We acknowledge that their exclusion may have introduced bias, particularly in terms of geographic or thematic representativeness. This left 221 articles for detailed review, from which 122 studies met the final inclusion criteria, comprising 119 empirical studies and 3 theoretical works (see Fig. 2). We provide a table in the supplementary material listing retrieved and not accessible articles.

Authorship patterns across the reviewed studies were highly variable, with the number of authors ranging from 1 to 15 per paper and an average of 6 authors (± 11.4). Institutional involvement was similarly diverse, with an average of 3.4 institutions (± 4.6) contributing to each study. Among the different mycobacterial groups, TB research exhibited the highest level of institutional collaboration, averaging 6.1 institutions per article (± 10.4), followed by studies on MU/BU, which averaged 4.8 institutions per study (± 8.7). Geographically, research on animal and human mycobacteria was conducted across 46 nations.

**Fig. 3:**
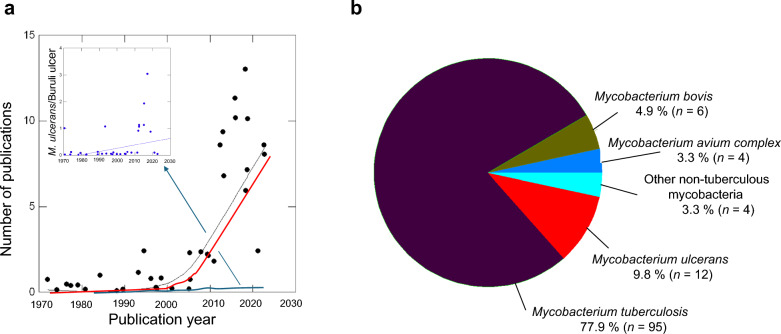
Bibliometric demographics on seasonal trends in mycobacterial infections. **a** Number of publications on mycobacterial infections published in 1971–2023 period. Total number of publications illustrated with black curve; number of publications on TB with red curve, and number of publications on MU/BU with blue curve (For scale reason, inlet corresponds to MU/BU scientific production in time). **b** Percentile breakdown by categories of mycobacterial infections excluding *M. leprae* article (*n* = 1). *TB* Tuberculosis, *MU Mycobacterium ulcerans*, *BU* Buruli ulcer

**Fig. 4:**
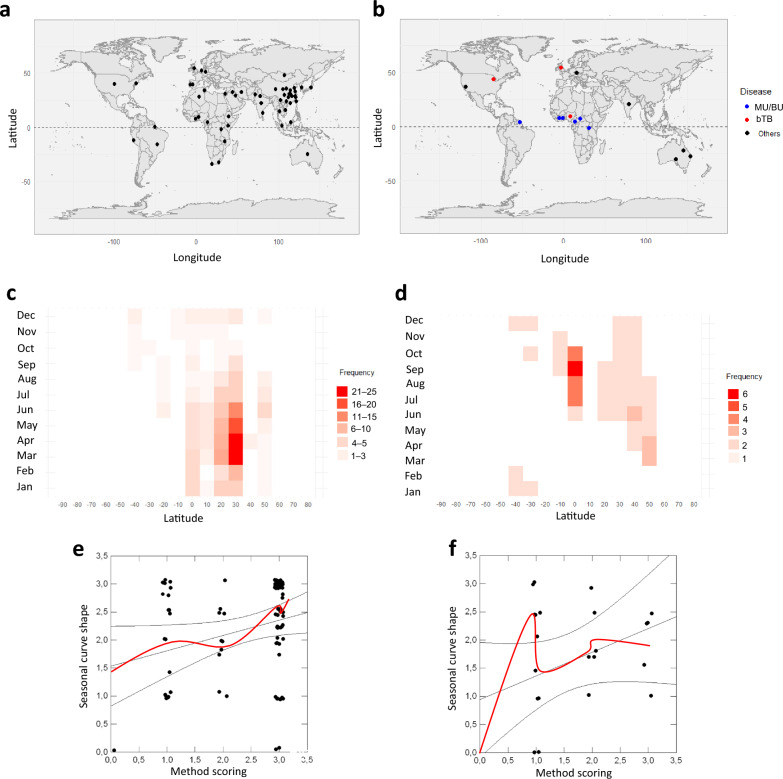
Distribution maps, seasonal trends and seasonal peak shapes for the different publications retained in our mixed-methods review. Upper panel: distribution map of research studies for **a** TB; and **b** other mycobacteria (except TB), including bTB and MU/BU. Blue dots: MU/BU, red dots bTB, and black dots others. Base map source: Country borders are based on the CIA World DataBank II. Middle panel: heatmap of the relationship between latitude (in degree) of a given study and month-s for which it is observed an epidemic peak for **c** TB studies; and **d** other mycobacterial diseases*.* Colors from white to red indicate publication frequency with white corresponding to no publication. Lower panel: relationship between statistical-mathematical methodology used (see Table 1) and curve shape of epidemic peak for **e** TB studies (*n* = 100, *F* = 6.9, *P* < 0.01); and **f** other mycobacteria studies (*n* = 25, *F* = 3.5, *P* = 0.07). The red curve illustrates the LOWESS tendency, with tension equals 0.5. TB Tuberculosis, MU Mycobacterium ulcerans, BU Buruli ulcer, bTB Bovine tuberculosis, *LOWESS* Locally Weighted Scatterplot Smoothing

The review of scientific articles on seasonal trends in mycobacterial infections from 1971 to 2023 revealed a clear historical progression. Early studies on the topic emerged in the 1970s, with limited publications until the early 2000s, when research activity significantly increased (Fig. 3a). This growth was particularly notable from 2010 to 2020, during which 10.1% (*n* = 12) of the reviewed articles were published. The number of studies per mycobacterial species or categories ranged from 1 to 93 and is shown in Fig. 3b. Geographical distribution of study locations are represented in Figs. 4a-b.

### Tuberculosis

Seasonal trends in human TB were most pronounced between 20° and 30° latitude North (Fig. 4c, with peak periods typically occurring from March to June. TB research accounted for 77.9% of the reviewed articles (*n* = 95). Graphical representation of peak frequencies confirmed that cases predominantly showed their acme in April/May annually.

China emerged as a leading contributor to TB seasonality research, producing 40.2% (*n* = 39) of the 93 publications. These studies predominantly applied advanced methodologies: Generalized Linear Models (GLMs) (*n* = 23, 18.5%), (Seasonal) AutoRegressive Integrated Moving Average (SARIMA) models (*n* = 22, 18%), and (Partial) Autocorrelation Functions (PACF/ACF) (*n* = 19, 15.6%). Articles using these techniques were more likely to identify pronounced seasonal patterns (type two or three cyclicity) than those relying on descriptive statistics or simple visual inspection.

### *Mycobacterium ulcerans* and Buruli ulcer

Research on MU/BU accounted for 9.8% (*n* = 12) of the overall studies retained. Seasonal peaks for MU/BU were observed primarily between July and October, with a median peak in August with an interquartile range (IQR) of 1.75 months (Fig. 4d). Studies often employed an ecosystem-based approach, integrating human cases with environmental pathogen dynamics in tropical or sub-tropical regions, including Cameroon, Benin, Ghana and Victoria province, Australia.

Among MU/BU studies, four investigated seasonal patterns in human cases, while four explored mycobacterial dynamics in environmental habitats such as soil, water, and vegetation, or host carriers. Four studies analySed simultaneously BU epidemiology and MU environmental dynamics [15–18]. Although advanced methods like wavelet analysis (*n* = 5, 4%) and Fourier’s decomposition (*n* = 3, 2.4%) were occasionally used, the methodological rigor in MU/BU also represented a variety of methods.

### Bovine tuberculosis (*Mycobacterium bovis*)

Research on bTB was geographically diverse, with contributions from regions such as the UK, Ireland, New Zealand, and South Africa. Seasonal peaks for bTB primarily occurred in September, coinciding with agricultural and wildlife management practices.

bTB studies constituted 5.0% (*n* = 6) of the reviewed articles. Methodologically, 66.7% (*n* = 4) relied on descriptive statistics, while inferential and advanced time-series analyses were underutilized (*n* = 2). This limitation highlights the need for more robust methodologies to fully understand bTB seasonality and its environmental drivers.

### Other mycobacterial species

Studies on MAP and other NTMs represented 2.4% (*n* = 4) and 3.3% (*n* = 4) respectively. These studies primarily addressed environmental dynamics rather than direct epidemiological trends in hosts. Of the eight studies, 75% (*n* = 6) utilized descriptive statistics, with only two applying advanced time-series techniques. Seasonal patterns for these mycobacteria were less defined due to the limited dataset in animal diseases in general, and simpler methodologies used. A single study analysing *M. leprae* seasonality was retrieved, but it provided insufficient data and was excluded from further analysis [19].

### The use of appropriate methods to reveal seasonal trends in mycobacterial infections

Among the 122 selected papers, the most frequently employed methodological tools were Generalized Linear Models and related models (GLMs) (*n* = 23, 18.5%), SARIMA (*n* = 22, 18%), and autocorrelation functions (ACF/PACF) (*n* = 19, 15.6%) (see Table 1 for details). Descriptive statistics were used in 18 studies (14.7%), and inferential statistics in 16 studies (13.1%). LOWESS was applied in 9 studies (7.4%), wavelet analysis in 5 studies (4%), differential equation-based models in 4 studies (3.3%), and Fourier decomposition in 3 studies (2.4%) [11].

**Table 1:** Categories of the methodology used to analyse seasonality per mycobacterial categories across the different selected papers

Method	Number of articles per mycobacterial group						Total number of articles
	*M.* *tuberculosis/* TB	*M. avium paratuberculosis*/MAP	Non-tuberculous mycobacteria/NTM	*M. bovis/*bTB	*M. ulcerans/*MU	*M. leprae/*leprosy	
Descriptive statistics	8	0	0	2	6	0	16
Inferential statistics	8	0	0	1	3	1	13
Heatmap	1	0	0	0	0	0	1
ANOVA	0	1	2	0	0	0	3
GLM	10	1	0	1	2	0	14
LOWESS	8	0	0	1	0	0	9
MEM	2	1	0	0	0	0	3
Fourier's analysis	3	0	0	0	0	0	3
Multiplicative decomposition	1	0	0	0	0	0	1
ACF/PACF	19	0	0	0	0	0	19
ARIMA/SARIMA	21	0	0	0	0	0	21
Wavelet analysis	3	0	0	0	1	0	4
Mathematical modelling	12	0	2	1	0	0	16

*ANOVA* Analysis of Variance, *MEM* Maximum Entropy Method, *LOWESS* Locally Weighted Scatterplot Smoothing, *GLM* Generalized Linear Model, *ACF/PACF* AutoCorrelation Function/Partial AutoCorrelation Function, *ARIMA/SARIMA* AutoRegressive Integrated Moving Average/Seasonal ARIMA

The methodology score averaged 1.86 (± 0.7) across all articles included in this review. This score reflects the level of methodological rigor in each study, ranging from descriptive approaches to more advanced statistical and time-series methods. Articles using descriptive statistics, which provided basic summaries of seasonal patterns, received the lowest average score (1.0 ± 0.01). Studies employing inferential methods, which assessed seasonality using statistical tests, had a slightly higher average score (1.5 ± 0.1). Articles utilizing advanced time-series methods, and which modelled temporal trends in detail, achieved the highest average score (2.4 ± 0.2). These scores indicate a direct relationship between the sophistication of the methodology and the capacity to identify and analyse seasonal trends in these mycobacterial infections.

### Research co-citation networks on mycobacteria and main tendencies

This study revealed two key findings from co-citation networks: First, China accounted for 40.6% (39 articles) of the 93 global publications on TB seasonality, highlighting its dominant role in this research area. Second, research on MU and BU adopted a comprehensive ecosystem-based approach, examining human cases and the dynamics of the environmental pathogen in aquatic animals, plants, soil and water together to better understand transmission at the interface [20]. Unlike other mycobacterial studies, MU/BU research frequently combined environmental and clinical data [15].

**Fig. 5:**
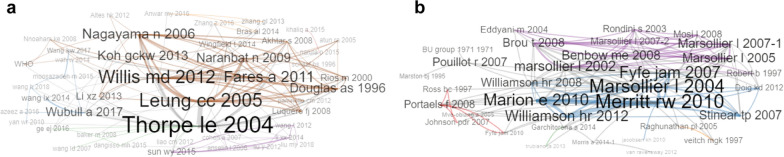
Co-citation networks of scientific research production on mycobacterial seasonality. **a** for Chinese research on TB, and **b** for MU/BU internationally. Co-citation network of the included articles. Node size is proportional to the weighted degree of each item (the sum of co-citations with all other items). Edge thickness is proportional to the frequency of co-citations between two items. The structure reflects the conceptual clustering of the literature. Items located near the center, exhibit higher centrality and lower farness, suggesting that they play a key role in connecting different clusters. TB Tuberculosis, MU* Mycobacterium ulcerans*, BU Buruli ulcer

Bibliometric networks identified influential studies within TB and MU/BU research. Thorpe et al. (2004) [21] achieved the highest PageRank (0.05) and betweenness (377.9) scores in TB research (Fig. 5a), followed by Leung et al. (2005) [22], Willis et al. (2012) [23], Nagayama et al. (2006) [24], and Fares (2011) [25]. For MU/BU, Merritt et al. (2010) [26] held the highest PageRank (0.05) among 47 nodes (Fig. 5b). Other notable contributors included Marion et al. (2010) [27] and Marsollier et al. (2004) [28], though statistical analyses to substantiate seasonality were most often absent. The network’s characteristics and scores can be consulted in supplementary materials.

### Research and seasonal trends

Collapsing data across all study years, our analysis demonstrated that the annual seasonal component was particularly prominent between 20° and 30° latitude North for TB (Fig. 4c), with a concentration of studies in these regions. The annual peak period for TB primarily occurred between March and June. For other mycobacteria, including MU associated with BU cases, seasonal trends were driven by research in tropical regions, where cases predominantly peaked between July and October. However, while BU cases peak during this period, MU itself appears to peak earlier. MU tends to peak 2 to 3 months before BU cases, suggesting a temporal lag between environmental MU prevalence and the onset of BU in humans. bTB exhibited a similar seasonal peak, with cases most frequently observed in September. These patterns were consistent across the studies included in this review.

Our analysis revealed significant geographical biases in the selected studies, with limited research focusing on seasonal trends in regions such as the southern hemisphere and areas above 40° North latitude in the northern hemisphere. Among the 122 papers documenting at least one epidemic peak per year, the mean peak period for all mycobacterial infections combined was from April to May, with a median in May and a standard deviation (SD) of ± 2.9 months, IQR equals to 3.5 months. TB exhibited a mean and median peak in May (IQR = 2.5 months), whereas BU and other mycobacterial diseases showed peak periods in August and September, respectively.

Using a four-category scale assessing epidemic peak amplitude shape (see Panel 1 and Fig. 1), the overall mean value was 2 (± 0.9) for all studies. Concerning TB studies, the mean value was 2.1 (± 0.6). MU/BU exhibited a mean of 2.6 (± 0.7), also therefore with a slightly higher score in disease amplitude. The Cohen' Kappa test (*κ*) for the level of agreement for the two raters (see Panel 1) resulted in a *κ* value of 0.67 (*n* = 119, *z* = 12.3, *P* < 0.0001), indicating very substantial agreement in scoring (0.6–0.8 corresponds to strong agreement between raters).

### Interdisciplinarity and adequate skills to reveal seasonal dynamics

To assess the relationship between methodology and seasonal score in estimating seasonal curve shapes (see Panel 1), both parametric and non-parametric regression analyses were applied (Fig. 4e, f). Linear regression of seasonal curve shape against methodological score was highly significant (*r*^2^ = 0.3, *F* = 16.9, *P* < 0.0001, test for normality = 0.2,* P* < 0.0001) confirming that the tendency to demonstrate seasonal dynamics between the different retained studies depends largely on the degree of statistical or mathematical technical Sophistication of the study. In TB research (Fig. 4e), advanced statistical and mathematical techniques were predominantly used, particularly in Chinese publications, showcasing a wide range of robust methods (see Supplementary Material for details). For other mycobacteria, notably MU associated with BU (Fig. 4f), the trend was less pronounced due to the smaller number of studies but still demonstrated a generally positive direction. Employing advanced techniques from fields such as disease ecology, population dynamics, and physics enhances the ability to detect seasonality in mycobacterial dynamics, facilitating the quantitative identification of temporal changes in disease cases or environmental pathogen occurrences across diverse geographic scales.

## Discussion

Despite a historical interest in seasonal cycle phenomena of infectious disease dynamics [2, 29, 30], and a more recent interest with the COVID-19 pandemic, studies on the existence of seasonal trend in infectious diseases remain poorly studied [31]. For instance, seasonal flu is very emblematic of important research on the factors responsible for seasonal epidemics [32]. Several authors consider three kinds of explanations for challenges associated with studying disease seasonality [30]: (1) seasonality causes are multifactorial and context-dependent; (2) non-linearity in complex disease dynamics may scramble annual variation in disease case incidence; and (3) ecological interactions among different pathogens act on individual agent disease dynamics also disrupting the single dynamics. Research has also highlighted, through comparative analysis of longitudinal surveillance data of seasonal flu in a biogeographic framework, the importance of spatial and climate variability on seasonal disease dynamics [33, 34].

For many animal and human infectious agents, limited research on seasonal transmission dynamics exists, including the absence of coordinated surveillance systems in many countries and cultural perceptions in medical and veterinary fields that deny the existence of epidemic or epizootic seasonality. This mindset is exemplified in the research on mycobacterioses. The analysis of seasonality in TB must take into account the specific challenge posed by the disease's long and variable latency period. Unlike acute respiratory infections, where infection and symptoms are closely linked in time, TB may exhibit a delay of months or even years between infection and the onset of clinical symptoms [22]. This latency can obscure seasonal signals, as cases diagnosed in a given month may result from exposures that occurred in previous seasons [22]. Furthermore, TB surveillance systems capture a mixture of recently transmitted cases and reactivation cases from latent infections, which often deviate from seasonal trends. Additionally, diagnosis and reporting delays, common in TB surveillance, may shift the apparent peak of reported cases away from the true season of transmission. Taken together, these factors likely contribute to an underestimation or blurring of seasonal signals in TB, and should be considered when interpreting our findings.

However, the present mixed-methods review shows seasonality patterns in epizooties or epidemics of diverse animal and human mycobacterioses, respectively. There are two likely explanations to this: 1) recent statistical and mathematical technical contributions are needed to reveal annual periodicities in complex disease dynamic time-series; and 2) the extraordinary research growth, particularly by Chinese teams on human TB, which tends to indicate that the demonstration of disease seasonality is also research effort-dependent. We also highlighted the important work carried out on MU/BU population dynamics for which an integration of environmental and epidemiological research on human cases has gradually led to a change in paradigm in the way in which this mycobacterial infection is today interpreted [35, 36].

Seasonal variation has important influence on mycobacterial transmission and incidence in animals and humans. For instance, bTB transmission in herds intensifies during colder seasons due to factors like increased indoor activity, crowding, and reduced indoor ventilation [37]. Seasonal dynamics also impact host human immune responses to TB. Vitamin D deficiency, a disease associated with winter months that is produced by low exposure to sunlight, influences immune responses and can make the host prone to be infected by TB in human and also in animals [38, 39]. An intriguing hypothesis suggests that elevated CD4 T-cell levels during winter might delay TB symptom onset until spring in humans, and this mechanism explains the presence of incidence peaks during spring and summer [40]. In the case of herds such as cattle, common pasture fields, water use and proximity to reservoir populations has a significant influence on bTB seasonal infections [41]. Phenological cycles, including, animal reproduction, further influence host reservoir behaviour. For instance, the heightened incidence of bTB among badgers during spring aligns with their reproductive and breeding phases, subjecting individuals to gathering and physiological stress, thus impacting bTB cases incidence and dynamics in cattle [42, 43]. Thus, understanding bTB seasonal dynamics requires an integrated approach that need an ecosystem-based, disease ecology orientated perspective [36]. We have emphasized that controlling bTB’s seasonality is not only an agricultural or veterinary issue but also a public health priority, highlighting the interconnected nature of disease management across species. Finally, host genetic factors – including genetic susceptibility – may modulate seasonal risk. For instance, populations with varying genetic predispositions (such as, in immune response or vitamin D metabolism) could experience different seasonal impacts, though direct evidence for genetic influences on mycobacterial disease seasonality is currently limited.

Environmental conditions also play a pivotal role in mycobacterial dynamics. In tropical regions, TB spread follows wet seasons, leading to fluctuating trends [25]. Collectively, these examples of TB, MU/BU and bTB, along with the findings of this present review suggest that seasonality is likely a common occurrence for most mycobacterial diseases, especially NTMs documented to be animal or human pathogens thriving in natural aquatic or terrestrial environments outside vertebrate hosts [44]. Beyond seasonality, additional factors strongly may influence mycobacterial epidemiology. Seasonal variations in human activities impact infection rates, as seen in agricultural harvesting periods, which affect TB incidence in many regions globally. [45]. Similarly, seasonal mass gatherings, such as pilgrimages, facilitate increased transmission of TB among travelers, impacting on seasonal epidemiology under favorable conditions [46]. In livestock, bTB infections within herds are also affected by interactions with non-domestic animals like badgers and wild boars, as well as factors like housing type and cattle exploitation practices [47].

Concerning MU population dynamics in aquatic ecosystems, they depend on the arrival of rainfall in tropical zones and vary with seasonal changes and variation in macroinvertebrate species population biology, which is closely linked to the arrival of the rains. There have been reported seasonal increases in MU environmental loads preceding BU cases by 2 to 3 months and suggesting an environmental route of transmission associated with seasonal rainfall patterns [35]. Also, experimental evidence indicates lower bTB soil concentrations during warmer seasons (specifically, spring/summer) due to reduced humidity, soil moisture and increased solar radiation [48, 49]. NTMs are known to be adaptable to diverse climates and habitat conditions [44], exhibit varying environmental concentrations throughout the year subject to seasonal changes [50, 51], water temperature in the supply network is related to human cases of diverse NTM species [52]. Climate shifts and land use and modifications influence MU presence in natural aquatic ecosystems, leading to runoff during heavy rainfall and soil erosion [53, 54], thus exposing more people and wild and domestic animals to this family of pathogens [55].

Studies on TB seasonality primarily concentrated on temperate northern regions. This geographical bias is noteworthy given that tropical populations are both most affected by TB and BU, and also the most vulnerable [56]. The same spatial bias was also observed for all other NTMs [3]. Many regions above 40° North and South latitudes, including Subarctic areas of North America and Eurasia, have lacked substantial research both in past years and currently. Seasonal trends studies for MU/BU have been concentrated in equatorial and tropical zones where the mycobacterial agent is prevalent, but only for the northern hemisphere except Australia, Papua-New Guinea and several other rare countries. This is due in part to the distribution of land masses that are more important in the northern hemisphere, and strong Western interest in research into neglected tropical diseases. To mitigate disease risks, a concerted global effort is needed to uncover seasonality patterns in mycobacterial infections in both animals and humans, especially in underrepresented countries and in the global South. This is more important as these regions are currently suffering the most from the morbid and lethal burden of these mycobacterial infections, both human and those affecting wild and farmed animals. This entails improving regional and global disease surveillance systems [57]. In addition, it also shows that short-term and fine-scale research on mycobacterial infections constitutes the rule for much research, and that efforts must be made over the longer term to reveal disease patterns and determinants which intervene on medium- and long-term scales [58].

Although both BU and TB exhibit seasonal trends, their underlying drivers are distinct. BU incidence often peaks during or after rainy seasons in endemic areas​ [17], reflecting environmental exposure (including aquatic reservoirs and insect vectors), whereas TB generally peaks in spring/summer​ due to factors like indoor crowding in winter and seasonal changes in host immunity [25]. We have highlighted this distinction to avoid suggesting a direct ecological similarity between BU/MU and TB.

A notable feature of the reviewed literature is the predominance of studies from China, particularly regarding TB. This predominance can be mainly attributed to China's combination of a high disease burden, the implementation of a comprehensive national surveillance system (TBIMS) since 2005 [59], and substantial investment in scientific research [60]. China possesses one of the largest TB surveillance datasets worldwide, allowing researchers to explore temporal trends such as seasonality in depth, both nationally and provincially. This research environment has positioned China as a leading contributor to the global TB literature [60]. Consequently, the overrepresentation of Chinese studies in our review likely reflects this country's data availability and research capacity, rather than fundamental biological differences in the seasonal behavior of TB compared to other regions. Considering that the Beijing genotype is one of the most important and virulent strains of TB, it is very possible that it could provoke more pronounced epidemic waves in populations that are also more sensitive to this strain, thus causing more marked seasonality [61, 62]. However, there is little evidence in the current international literature, and this should therefore constitute a new area of research for the future. Co-citation networks showed that research groups in China primarily relied on disease temporal-series research conducted in other countries. They used milestone Western publications, such as Leung and collaborators, as a reference to apply advanced statistical methods for demonstrating TB seasonality in various states and regions [22]. The case of research tendency on MU/BU indicates a relatively new interdisciplinary approach, more precisely disease ecology that adopts an ecosystem-based approach [63]. Epidemiological perspectives were dominant in the XX^th.^ century until 2015, followed then by a strong collaboration of other fields such as disease ecology and population dynamics. The recent literature on MU/BU is largely based on articles that only textually suggested seasonal trends in disease and pathogen occurrence but did not statistically test for such patterns. Nonetheless, current studies employing advanced statistical and mathematical tools have advanced our knowledge in MU/BU time-series analysis that can be used for many other mycobacterial or non-mycobacterial diseases [15, 17, 64]

A limitation of our review is that some publications may not have been indexed in various bibliographic databases, especially from lower-income and developing countries. Notably, several high-TB-burden regions – for example, South Asia (India, Pakistan, and others) and sub-Saharan Africa – had very few studies on seasonality. One possible explanation is that countries near the Equator experience less pronounced seasonality in TB incidence​, making seasonal patterns more subtle. Additionally, there may be a publication or research bias regarding the availability of information: limited surveillance data, fewer studies undertaken, or challenges in indexing studies from these regions.

Our review was restricted to articles published in English, French, or Spanish, which may have led to a language bias by excluding relevant studies published in other languages, such as Portuguese, Russian, Chinese, or local African and Asian languages. This limitation may have contributed to the underrepresentation of certain regions in our analysis, particularly countries from South America, Central Asia, or parts of sub-Saharan Africa. As noted by other authors, language restrictions can influence systematic reviews by narrowing the geographical diversity of included studies [65]. We acknowledge that this could limit the generalizability of our conclusions regarding global seasonality patterns of mycobacterial diseases. Further, we acknowledge that our review did not include a ‘control’ disease for baseline comparison. Including a disease known to have minimal seasonal variation (as a negative control) in future analyses could help distinguish general seasonal effects from disease-specific patterns. We recommend this approach for future studies to strengthen the attribution of observed seasonality to the infection itself.

## Conclusions

In our mixed-methods review, we emphasize the significance of geographic and technical considerations in publications assessing seasonal dynamics of animal and human mycobacterial diseases. Existing reviews have mainly focused on human TB, reporting seasonal trends [25, 66, 67], while the broader picture of seasonal patterns in mycobacterial diseases, both in animals and humans, remains not explored due to limited longitudinal data. In this comparative study, we demonstrate that seasonal dynamics are important in most of animal and human mycobacterioses, with most of the research still focusing on TB. Other diseases, such as MU/BU in humans and bTB disease in herds and wildlife, tend also to show a regular seasonal pattern.

The underrepresentation of research in the southern hemisphere is glaring and should be of global health concern, as we could expect a 6-month time lag with the northern hemisphere. Concerning MU/BU, studies were logically concentrated in tropical areas where the disease is prevalent. Furthermore, we observe two new important trends in recent research on this topic that can be generalized to most infectious diseases. First, our study demonstrates that the use of appropriate statistical and mathematical tools, from disciplines other than that of human and veterinary medicine and microbiology, are essential to understanding mycobacterial disease seasonality; therefore, requiring more interdisciplinarity and transdisciplinarity, with the inclusion of other approaches and concepts in than those largely used in medicine and microbiology. Second, the example of MU/BU is an example of how an ecosystem-based framework to studying human and animal disease dynamics can help improve human capacity for study, surveillance, and scientific and medical infrastructure to better prepare and strengthen the global mycobacterial health response to global change threats.

Going forward, this review also poses the more epistemological subject on the scientific method in human and veterinary medicine, namely that a given pattern does not exist because it has not been demonstrated. Certainly, the existence of seasonal dynamics among animal and human mycobacterial diseases should be accepted as the rule and be considered as a plausible hypothesis against which one seeks to demonstrate that it is not verified according to such geographical, environmental or socio-economic conditions. Better understanding disease ecology and population dynamics in space and time will enable animal and human health organizations and public health agencies to better support wildlife, livestock, and human communities in a current context of regional and global environmental and demographic changes.

## Availability of data

Articles and Co-citation network scores in supplementary materials.

## Supplementary Information

Supplementary Material 1.

Supplementary Material 2.

Supplementary Material 3.

## Data Availability

The data analysed during the current study are available in the supplementary materials associated with this article. Additional information or clarifications can be obtained from the corresponding author upon reasonable request.

## Abbreviations

ACF/PACF: AutoCorrelation Function / Partial AutoCorrelation Function
ARIMA/SARIMA: (Seasonal) AutoRegressive Integrated Moving Average
bTB: Bovine tuberculosis
BU: Buruli ulcer
GLM: Generalized Linear Model
IQR: Interquartile range
LOWESS: Locally Weighted Scatterplot Smoothing
MAP: *Mycobacterium avium paratuberculosis/*Johne’s disease
MTBC: *Mycobacterium tuberculosis* complex
MU: *Mycobacterium ulcerans*
NTM: Non-tuberculous mycobacteria
PRISMA: Preferred Reporting Items for Systematic Reviews and Meta-Analyses
SD: Standard deviation
TB: Tuberculosis
TBIMS: Tuberculosis Information Management System
