# Supplementary material for: Seasonality and mycobacterial infectious diseases in animals and humans: is there a generality of seasonal patterns for mycobacterial infections?
Source: Infect Dis Poverty. 2025 Jul 3;14:59. doi: 10.1186/s40249-025-01319-3 (PMC12225213; doi:10.1186/s40249-025-01319-3)
Supplement: Supplementary file 1 — Supplementary Material 1. [file 40249_2025_1319_MOESM1_ESM.docx]

Supplementary material

Content

[Search script 1](#_Toc197525935)

[Bibliography retained in this review 2](#_Toc197525936)

[Articles not retrieved 8](#_Toc197525937)

[Representative articles for quality assessment classification 10](#_Toc197525938)

# Search script

We performed a systematic search in three major bibliographic databases: PubMed-Medline™, Scopus™, and Web of Science™ (Clarivate Analytics) covering the period from January 1st, 1971 to April 7th, 2023. The search strings combined terms related to mycobacterial species and seasonality, using Boolean operators and truncation. Below we detail the complete search strategies used for each database.

In PubMed, we used the following search string:

("Mycobacterium ulcerans" OR "Non-tuberculous mycobacteria" OR "Buruli ulcer" OR "Mycobacter*" OR "Mycobacterium tuberculosis" OR "tuberculosis") AND ("Season*" OR "climate trend" OR "climate pattern" OR "Periodicity" OR "seasonality" OR "seasonal trend*" OR "seasonal pattern*" OR "climate-driven" OR "seasonal variation" OR "seasonal demonstration" OR "seasonal evidence" OR "seasonal cycles" OR "seasonal cycling" OR "seasonal periodicity" OR "Climate variability" OR "Weather pattern*" OR "Climatic fluctuation*" OR "Atmospheric cycle" OR "Meteorological trend*" OR "Environmental rhythm*" OR "Climate oscillation*" OR "Atmospheric dynamic*" OR "Climatic change*" OR "Weather cycle*").

In Scopus, the search was conducted using:

TITLE-ABS-KEY("Mycobacterium ulcerans" OR "Non-tuberculous mycobacteria" OR "Buruli ulcer" OR "Mycobacter*" OR "Mycobacterium tuberculosis" OR "tuberculosis") AND TITLE-ABS-KEY("Season*" OR "climate trend" OR "climate pattern" OR "Periodicity" OR "seasonality" OR "seasonal trend*" OR "seasonal pattern*" OR "climate-driven" OR "seasonal variation" OR "seasonal demonstration" OR "seasonal evidence" OR "seasonal cycles" OR "seasonal cycling" OR "seasonal periodicity" OR "Climate variability" OR "Weather pattern*" OR "Climatic fluctuation*" OR "Atmospheric cycle" OR "Meteorological trend*" OR "Environmental rhythm*" OR "Climate oscillation*" OR "Atmospheric dynamic*" OR "Climatic change*" OR "Weather cycle*").

In Web of Science, we used:

TS=("Mycobacterium ulcerans" OR "Non-tuberculous mycobacteria" OR "Buruli ulcer" OR "Mycobacter*" OR "Mycobacterium tuberculosis" OR "tuberculosis") AND TS=("Season*" OR "climate trend" OR "climate pattern" OR "Periodicity" OR "seasonality" OR "seasonal trend*" OR "seasonal pattern*" OR "climate-driven" OR "seasonal variation" OR "seasonal demonstration" OR "seasonal evidence" OR "seasonal cycles" OR "seasonal cycling" OR "seasonal periodicity" OR "Climate variability" OR "Weather pattern*" OR "Climatic fluctuation*" OR "Atmospheric cycle" OR "Meteorological trend*" OR "Environmental rhythm*" OR "Climate oscillation*" OR "Atmospheric dynamic*" OR "Climatic change*" OR "Weather cycle*").

# Bibliography retained in this review

1. Abdul IW, Ankamah S, Iddrisu AK, Danso E. Space-time analysis and mapping of prevalence rate of tuberculosis in Ghana. *Scientific African.* 2020;**7**: e00307.
2. Abebe G, Zegeye Bonsa WK. Seasonal behavior and forecasting trends of tuberculosis incidence in Holy Kerbala, Iraq. International Journal of Mycobacteriology. 2017;**6(3):**239–245.
3. Aboagye SY, Ampah KA, Ross A, Asare P, Otchere ID, Fyfe J, Yeboah-Manu D. Seasonal Pattern of *Mycobacterium ulcerans*, the Causative Agent of Buruli Ulcer, in the Environment in Ghana. *Microbial Ecology*. 2017;**74(2):**350–361.
4. Ade S, Békou W, Adjobimey M, Adjibode O, Ade G, Harries AD, Anagonou S. Tuberculosis Case Finding in Benin, 2000–2014 and Beyond: A Retrospective Cohort and Time Series Study. *Tuberculosis Research and Treatment*. 2016;**2016**:1–9.
5. Aissa S, Maoua M, Benzarti W, Gargouri I, Lassoued R, Sfaxi R, et al. Seasonality of Pulmonary Tuberculosis in Sousse (Tunisia). *Tunis Med*. 2019 Jun;**97(6)**:808–17. (in #French#)
6. Alene KA, Xu Z, Bai L, Yi H, Tan Y, Gray DJ, Viney K, Clements ACA. Spatiotemporal patterns of tuberculosis in Hunan province, China. International *Journal of Environmental Research and Public Health.* 2021;**18(13):** 6778.
7. Amofah GK, Adjei-Acquah C, Sagoe-Moses C, Frimpong EH. Epidemiology of buruli ulcer in amansie west district, Ghana*. Transactions of the Royal Society of Tropical Medicine and Hygiene.* 1993;**87(6)**:644–645.
8. Andrews JR, Cobelens F, Horsburgh CR, Hatherill M, Basu S, Hermans S, Wood R. Seasonal drivers of tuberculosis: Evidence from over 100 years of notifications in Cape Town. *International Journal of Tuberculosis and Lung Disease*. 2020;**24(5)**:477–484.
9. Ane-Anyangwe IN, Akenji TN, Mbacham WF, Penlap VN, Titanji VPK. Seasonal variation and prevalence of tuberculosis among health seekers in the South Western Cameroon. *East African Medical Journal.* 2006;**83(11**):588–595.
10. Assan B, Nyabadza F, Landi P, Hui C. Modeling the transmission of Buruli ulcer in fluctuating environments. *International Journal of Biomathematics*. 2017;**10(5):** 1750063.
11. Azeez A, Obaromi D, Odeyemi A, Ndege J, Muntabayi R. Seasonality and trend forecasting of tuberculosis prevalence data in Eastern Cape, South Africa, using a hybrid model. *International Journal of Environmental Research and Public Health*. 2016;**13(8);**757**.**
12. Bonell A, Contamin L, Thai PQ, Thuy H. T. T., Van Doorn HR, White R, Nadjm B, Choisy M. Does sunlight drive seasonality of TB in Vietnam? A retrospective environmental ecological study of tuberculosis seasonality in Vietnam from 2010 to 2015. *BMC Infectious Diseases.* 2020;**20(1):**1–11.
13. Bowong S, Kurths J. Modeling and parameter estimation of tuberculosis with application to Cameroon. International *Journal of Bifurcation and Chaos.* 2011;**21(7):**1999–2015.
14. Bowong S, Kurths J. Modeling and analysis of the transmission dynamics of tuberculosis without and with seasonality. *Nonlinear Dynamics.* 2012;**67(3)**:2027–2051.
15. Bras AL, Gomes D, Filipe PA, De Sousa B, Nunes C. Trends, seasonality and forecasts of pulmonary tuberculosis in Portugal. *International Journal of Tuberculosis and Lung Disease.* 2014;**18(10)**:1202–1210.
16. Butt MF, Younis S, Wu Z, Hadi SH, Latif A, Martineau AR. The relationship between seasonality, latitude and tuberculosis notifications in Pakistan. *BMC Infectious Diseases*. 2021;**21(1)**:21–27.
17. Cao K, Yang K, Wang C, Guo J, Tao L, Liu Q, Gehendra M, Zhang Y, Guo X. Spatial-temporal epidemiology of tuberculosis in mainland China: An analysis based on Bayesian theory. International *Journal of Environmental Research and Public Health*. 2016;**13(5)**:4–8.
18. Cao S, Wang F, Tam W, Tse LA, Kim JH, Liu J, Lu Z. A hybrid seasonal prediction model for tuberculosis incidence in China. *BMC Medical Informatics and Decision Making.* 2013;**13(1)**:56.
19. Carpenter TE, Hird DW. Time series analysis of mycobacteriosis in California slaughter swine. *Preventive Veterinary Medicine.* 1986**;3(6):**559–572.
20. Charles T, Eckardt M, Karo B, Haas W, Kröger S. Seasonality in extra-pulmonary tuberculosis notifications in Germany 2004-2014- a time series analysis. *BMC Public Health.* 2021;**21(1):**1–9.
21. Chaw L, Liew SQR, Wong J. Association between climate variables and pulmonary tuberculosis incidence in Brunei Darussalam. *Scientific Reports*. 2022;**12(1)**:1–9.
22. Chen J, Qiu Y, Yang R, Li L, Hou J, Lu K, Xu L. The characteristics of spatial-temporal distribution and cluster of tuberculosis in Yunnan Province, China, 2005-2018. *BMC Public Health.* 2019;**19(1)**:1–13.
23. Chen MP, Shang N, Winston CA, Becerra JE. A Bayesian analysis of the 2009 decline in tuberculosis morbidity in the United States. *Statistics in Medicine*. 2012;**31(27):**3278–3284.
24. Chinpong K, Thavornwattana K, Armatrmontree P, Chienwichai P, Lawpoolsri S, Silachamroon U, Maude RJ, Rotejanaprasert C. Spatiotemporal Epidemiology of Tuberculosis in Thailand from 2011 to 2020. *Biology*. 2022;**11(5):**1–14.
25. Combe M, Gozlan RE, Jagadesh S, Velvin CJ, Ruffine R, Demar MP, Couppié P, Djossou F, Nacher M, Epelboin L. Comparison of *Mycobacterium ulcerans* (Buruli ulcer) and Leptospira sp. (Leptospirosis) dynamics in urban and rural settings. *PLoS Neglected Tropical Diseases.* 2019;**13(1):**1–18.
26. Cui Z, Lin D, Chongsuvivatwong V, Zhao J, Lin M, Ou J, Zhao J. Spatiotemporal patterns and ecological factors of tuberculosis notification: A spatial panel data analysis in Guangxi, China. *PLoS ONE.* 2019;**14(5):**1–15.
27. de Andrade HLP, Gomes D, Ramos ACV, Arroyo LH, Santos-Neto M, Palha PF, Fiorati RC, Fronteira I, Monroe AA, dos Santos MS, Fuentealba-Torres M, Yamamura M, Crispim J de A, Arcêncio RA. Tuberculosis forecasting and temporal trends by sex and age in a high endemic city in northeastern Brazil: where were we before the Covid-19 pandemic? *BMC Infectious Diseases.* 2021;**21(1):**1–10.
28. Delahay RJ, Walker N, Smith GS, Wilkinson D, Clifton-Hadley RS, Cheeseman CL, Tomlinson AJ, Chambers MA. Long-term temporal trends and estimated transmission rates for *Mycobacterium bovis* infection in an undisturbed high-density badger (Meles meles) population. *Epidemiology and Infection*. 2013;**141(7):**1445–1456.
29. Douglas AS, Strachan DP, Maxwell JD. Seasonality of tuberculosis: The reverse of other respiratory diseases in the UK. *Thorax*. 1996;**51(9):**944–946.
30. Duan Y, Cheng J, Liu Y, Fang Q, Sun M, Cheng C, Han C, Li X. Epidemiological Characteristics and Spatial-Temporal Analysis of Tuberculosis at the County-Level in Shandong Province, China, 2016–2020. *Tropical Medicine and Infectious Disease*. 2022;**7(11):**346.
31. Fares A. Seasonality of tuberculosis. *Journal of Global Infectious Diseases.* 2011;3(1):46–55.
32. Fernandes FMC, Martins ES, Pedrosa DMAS, Evangelista MSN. Relationship between climatic factors and air quality with tuberculosis in the Federal District, Brazil, 2003–2012. *Brazilian Journal of Infectious Diseases.* 2017;**21(4)**:369–375.
33. Fine AE, Bolin CA, Gardiner JC, Kaneene JB. A study of the persistence of *Mycobacterium bovis* in the environment under natural weather conditions in Michigan, USA. *Veterinary Medicine International.* 2011;**2011**: 765430.
34. Garchitorena A, Guégan J-F, Léger L, Eyangoh S, Marsollier L, Roche B. *Mycobacterium ulcerans* dynamics in aquatic ecosystems are driven by a complex interplay of abiotic and biotic factors. *ELife.* 2015;**4**:1–19.
35. Garchitorena, A., Ngonghala, C. N., Texier, G., Landier, J., Eyangoh, S., Bonds, M. H., Guégan, J. F., & Roche, B. . Environmental transmission of *Mycobacterium ulcerans* drives dynamics of Buruli ulcer in endemic regions of Cameroon. *Scientific Report*s. 2015 5.
36. Ge E, Zhang X, Wang X, Wei X. Spatial and temporal analysis of tuberculosis in Zhejiang Province, China, 2009-2012. *Infectious Diseases of Poverty*. 2016;**5(1)**:1–10.
37. Gelaw Y, Yu W, Magalha R, Assefa Y, Williams G. Effect of temperature and altitude difference on tuberculosis notification: A systematic review. *Journal of Global Infectious Diseases.* 2019;**11(2):**63–68.
38. Ghadimi-Moghadam A, Salahi M, Ghatee MA, Ghadimi-Moghadam A, Kanannejad Z, Mosavi A, Ramshk O, Khoramrooz S. Environmental and climatic factors influencing the occurrence and distribution of tuberculosis in southwest Iran: A GIS-based study. *Acta Medica Mediterranea*. 2020;**36(1)**:557–563.
39. Giacomet CL, Santos MS, Berra TZ, Alves YM, Alves LS, da Costa FBP, Ramos ACV, de Almeida Crispim J, Monroe AA, Pinto IC, Fiorati RC, Arcoverde MAM, Gomes D, de Freitas GL, Yamamura M, Arcêncio RA. Temporal trend of tuberculosis incidence and its spatial distribution in Macapá – Amapá. *Revista de Saude Publica*. 2021;**55**:1–12.
40. Guo C, Du Y, Shen SQ, Lao XQ, Qian J, Ou CQ. Spatiotemporal analysis of tuberculosis incidence and its associated factors in mainland China. *Epidemiology and Infection.* 2017;**145(12):**2510–2519.
41. Hennigan CE, Myers L, Ferris MJ. Environmental distribution and seasonal prevalence of *Mycobacterium ulcerans* in southern Louisiana. *Applied and Environmental Microbiology.* 2013;79(8):2648–2656.
42. Heuer C, Mitchell RM, Schukken YH, Lu Z, Verdugo C, Wilson PR. Modelling transmission dynamics of paratuberculosis of red deer under pastoral farming conditions. *Preventive Veterinary Medicine*. 2012;**106(1):**63–74.
43. Hu X. Threshold dynamics for a tuberculosis model with seasonality. *Mathematical Biosciences and Engineering.* 2012;**9(1):**111–122.
44. Huang L, Li XX, Abe EM, Xu L, Ruan Y, Cao CL, Li SZ. Spatial-temporal analysis of pulmonary tuberculosis in the northeast of the Yunnan province, *People’s Republic of China. Infectious Diseases of Poverty*. 2017;**6(1):**1–11.
45. Khaliq A, Batool SA, Chaudhry MN. Seasonality and trend analysis of tuberculosis in Lahore, Pakistan from 2006 to 2013*. Journal of Epidemiology and Global Health.* 2015;**5(4):**397–403.
46. Kim EH, Bae JM. Seasonality of tuberculosis in the Republic of Korea, 2006-2016. *Epidemiology and Health.* 2018;**40**:e2018051.
47. King HC, Murphy A, James P, Travis E, Porter D, Hung YJ, Sawyer J, Cork J, Delahay RJ, Gaze W, Courtenay O, Wellington EM. The variability and seasonality of the environmental reservoir of *Mycobacterium bovis* shed by wild European badgers. *Scientific Reports*. 2015;**5**:1–7.
48. Kirolos A, Thindwa D, Khundi ME, Burke RM, Henrion MYR, Nakamura I, Divala TH, Nliwasa M, Corbett EL, MacPherson P. Tuberculosis case notifications in Malawi have strong seasonal and weather-related trends. *Scientific Reports*. 2021;**11(1)**:1–9.
49. Koh GCKW, Hawthorne G, Turner AM, Kunst H, Dedicoat *M. tuberculosis* Incidence Correlates with Sunshine: An Ecological 28-Year Time Series Study. *PLoS ONE.* 2013;**8(3**):1–5.
50. Kohei Y, Sumi A, Kobayashi N. Time-series analysis of monthly age-specific numbers of newly registered cases of active tuberculosis in Japan from 1998 to 2013. *Epidemiology and Infection.* 2016;**144(11)**:2401–2414.
51. Korthals Altes H, Kremer K, Erkens C, Van Soolingen D, Wallinga J. Tuberculosis seasonality in the Netherlands differs between natives and non-natives: A role for vitamin D deficiency? *International Journal of Tuberculosis and Lung Disease.* 2012;**16(5):**639–644.
52. Krishnan R, Thiruvengadam K, Jayabal L, Selvaraju S, Watson B, Malaisamy M, Nagarajan K, Tripathy SP, Chinnaiyan P, Chandrasekaran P. An influence of dew point temperature on the occurrence of *Mycobacterium tuberculosis* disease in Chennai, India*. Scientific Reports*. 2022;**12(1)**:1–10.
53. Kuan MM. Applying SARIMA, ETS, and hybrid models for prediction of tuberculosis incidence rate in Taiwan. *PeerJ*. 2022;**10:** e13117.
54. Kubalek I, Komenda S. Seasonal variations in the occurrence of environmental mycobacteria in potable water. *Apmis*. 1995;**103(1–6)**:327–330.
55. 1. Kumar V, Singh A, Adhikary M, Daral S, Khokhar A, Singh S. Seasonality of Tuberculosis in Delhi, India: A Time Series Analysis. Perlman DC, editor. *Tuberc Res Treat* . 2014;**2014**:514093.
56. Landier J, de Magny GC, Garchitorena A, Guégan JF, Gaudart J, Marsollier L, Gall P Le, Giles-Vernick T, Eyangoh S, Fontanet A, Texier G. Seasonal patterns of buruli ulcer incidence, central Africa, 2002–2012. *Emerging Infectious Diseases.* 2015;**21(8)**:1414–1417.
57. Lau LHW, Wong NS, Leung CC, Chan CK, Lau AKH, Tian L, Lee SS. Seasonality of tuberculosis in intermediate endemicity setting dominated by reactivation diseases in Hong Kong. *Scientific Reports.* 2021;**11(1**):1–10.
58. Leung CC, Yew WW, Chan TYK, Tam CM, Chan CY, Chan CK, Tang N, Chang KC, Law WS. Seasonal pattern of tuberculosis in Hong Kong. *International Journal of Epidemiology.* 2005;**34(4):**924–930.
59. Li XX, Wang LX, Zhang H, Du X, Jiang SW, Shen T, Zhang YP, Zeng G. Seasonal Variations in Notification of Active Tuberculosis Cases in China, 2005-2012. *PLoS ONE.* 2013;**8(7)**:2005–2012.
60. Li Y, Zhu L, Lu W, Chen C, Yang H. Seasonal variation in notified tuberculosis cases from 2014 to 2018 in eastern China. *Journal of International Medical Research*. 2020;**48(8)**: 0300060520949031**.**
61. Li Z, Wang Z, Song H, Liu Q, He B, Shi P, Ji Y, Xu D, Wang J. Application of a hybrid model in predicting the incidence of tuberculosis in a Chinese population. *Infection and Drug Resistance.* 2019;**12**:1011–1020.
62. Liao CM, Hsieh NH, Huang TL, Cheng YH, Lin YJ, Chio CP, Chen SC, Ling MP. Assessing trends and predictors of tuberculosis in Taiwan. *BMC Public Health.* 2012;**12(1)**:29.
63. Liao Z, Zhang X, Zhang Y, Peng D. Seasonality and Trend Forecasting of Tuberculosis Incidence in Chongqing, China. Interdisciplinary Sciences – *Computational Life Sciences*. 2019;**11(1):**77–85.
64. Lin YJ, Liao CM. Seasonal dynamics of tuberculosis epidemics and implications for multidrug-resistant infection risk assessment. *Epidemiology and Infection*. 2014;**142**(2):358–370.
65. Lin Y, Liang D, Liang X, Huang M, Lin M, Cui Z. Space-Time Distribution Characteristics of Tuberculosis and Its Socioeconomic Factors in Southern China from 2015 to 2019. *Infection and Drug Resistance.* 2022;**15**(May):2603–2616.
66. Liu L, Zhao XQ, Zhou Y. A tuberculosis model with seasonality. *Bulletin of Mathematical Biology.* 2010;**72(4)**:931–952.
67. Liu Q, Li Z, Ji Y, Martinez L, Zia UH, Javaid A, Lu W, Wang J. Forecasting the seasonality and trend of pulmonary tuberculosis in Jiangsu Province of China using advanced statistical time-series analyses. *Infection and Drug Resistance.* 2019; **12:2311**–2322.
68. Luo T, Sumi A, Zhou D, Kobayashi N, Mise K, Yu B, Kong D, Wang J, Duan Q. Seasonality of reported tuberculosis cases from 2006 to 2010 in Wuhan, China. *Epidemiology and Infection.* 2014;**142(10):**2036–2048.
69. Luo Y, Degang Y, Ohtsuka M, Ishido Y, Ishii N, Suzuki K. Detection of *Mycobacterium ulcerans* subsp. shinshuense DNA from a water channel in familial Buruli ulcer cases in Japan. *Future Microbiology.* 2015;**10(4):**461–469.
70. Luquero FJ, Sanchez-Padilla E, Simon-Soria F, Eiros JM, Golub JE. Trend and seasonality of tuberculosis in Spain, 1996-2004. *International Journal of Tuberculosis and Lung Disease*. 2008;**12(2)**:221–224.
71. Mabaera B, Naranbat N, Katamba A, Laticevschi D, Lauritsen JM, Rieder HL. Seasonal variation among tuberculosis suspects in four countries*. International Health.* 2009;**1(1):**53–60.
72. MacLachlan JH, Lavender CJ, Cowie BC. Effect of Latitude on Seasonality of Tuberculosis, Australia, 2002-2011. *Emerging Infectious Diseases*. 2012; **18(11):**2011–2013.
73. Maharjan B, Gopali RS, Zhang Y. A scoping review on climate change and tuberculosis. *International Journal of Biometeorology.* 2021;**65(10):**1579–1595.
74. Manabe T, Takasaki J, Kudo K. Seasonality of newly notified pulmonary tuberculosis in Japan, 2007-2015. *BMC Infectious Diseases*. 2019;**19(1**):1–8.
75. Mao Q, Zhang K, Yan W, Cheng C. Forecasting the incidence of tuberculosis in China using the seasonal auto-regressive integrated moving average (SARIMA) model. *Journal of Infection and Public Health*. 2018;**11(5):**707–712.
76. Margalit I, Block C, Mor Z. Seasonality of tuberculosis in Israel, 2001-2011. *International Journal of Tuberculosis and Lung Disease.* 2016;**20(12)**:1588–1593.
77. Marion E, Eyangoh S, Yeramian E, Doannio J, Landier J, Aubry J, Fontanet A, Rogier C, Cassisa V, Cottin J, Marot A, Eveillard M, Kamdem Y, Legras P, Deshayes C, Saint-André JP, Marsollier L. Seasonal and regional dynamics of *M. ulcerans* transmission in environmental context: deciphering the role of water bugs as hosts and vectors. *PLoS Neglected Tropical Diseases.* 2010;**4(7)**: e731**.**
78. Mishra PS, Narang P, Narang R, Goswami B, Mendiratta DK. Spatio-temporal study of environmental nontuberculous mycobacteria isolated from Wardha district in Central India. Antonie van Leeuwenhoek, International *Journal of General and Molecular Microbiology*. 2017;**111(1):**73–87.
79. Moosazadeh M, Nasehi M, Bahrampour A, Khanjani N, Sharaf S, Ahmadi S. Forecasting tuberculosis incidence in Iran using box-Jenkins models. *Iranian Red Crescent Medical Journal*. 2014;**16(5)**: e11779**.**
80. Morrow RH, Pike MC, D.J. B. Epidemiology of *Mycobacterium ulcerans* infection (buruli ulcer) at Kinyara, Uganda. *Transactions of the Royal Society of Tropical Medicine and Hygiene*. 1971;**65(6)**:763–775.
81. Moustakas A, Evans MR. Regional and temporal characteristics of bovine tuberculosis of cattle in Great Britain. *Stochastic Environmental Research and Risk Assessment.* 2016;**30(3)**:989–1003.
82. Naranbat N, Nymadawa P, Schopfer K, Rieder HL. Seasonality of tuberculosis in an Eastern-Asian country with an extreme continental climate. *European Respiratory Journal.* 2009;**34(4)**:921–925.
83. Narula P, Sihota P, Azad S, Lio P. Analyzing seasonality of tuberculosis across Indian states and union territories. *Journal of Epidemiology and Global Health.* 2015;**5(4):**337–346.
84. Okeke LA, Fawole O, Muhammad M, Okeke IO, Nguku P, Wasswa P, Dairo D, Cadmus S. Bovine tuberculosis: a retrospective study at Jos abattoir, Plateau State, Nigeria*. The Pan African Medical Journal.* 2016;**25**:202.
85. Onozuka D, Hagihara A. The association of extreme temperatures and the incidence of tuberculosis in Japan. *International Journal of Biometeorology.* 2015;**59(8):**1107–1114.
86. Parrinello CM, Crossa A, Harris TG. Seasonality of tuberculosis in New York City, 1990-2007. *International Journal of Tuberculosis and Lung Disease*. 2012;**16(1)**:32–37.
87. Rao HX, Zhang X, Zhao L, Yu J, Ren W, Zhang XL, Ma YC, Shi Y, Ma BZ, Wang X, Wei Z, Wang HF, Qiu LX. Spatial transmission and meteorological determinants of tuberculosis incidence in Qinghai Province, China: A spatial clustering panel analysis. *Infectious Diseases of Poverty*. 2016;**5(1):**45**.**
88. Ríos M, García JM, Sánchez JA, Pérez D. A statistical analysis of the seasonality in pulmonary tuberculosis. *European Journal of Epidemiology.* 2000;**16(5)**:483–488.
89. Ruxton GD. The Effects of Stochasticity and Seasonality on Model Dynamics: Bovine Tuberculosis in Badgers*. The Journal of Animal Ecology*. 1996;**65(4)**:495.
90. Santos LG, Pires GN, Azeredo Bittencourt LR, Tufik S, Andersen ML. Chronobiology: Relevance for tuberculosis. *Tuberculosis.* 2012;**92(4)**:293–300.
91. Schehrazad S, Mohameed L. Spatiotemporal Analysis and Seasonality of Tuberculosis in Algeria Schehrazad. *International Journal of Mycobacteriology.* 2017;**6(3):**239–245.
92. Siamba S, Otieno A, Koech J. Application of ARIMA, and hybrid ARIMA Models in predicting and forecasting tuberculosis incidences among children in Homa Bay and Turkana Counties, Kenya. *PLOS Digital Health.* 2023;**2(2)**:e0000084.
93. Soetens LC, Boshuizen HC, Korthals Altes H. Contribution of seasonality in transmission of *Mycobacterium tuberculosis* to seasonality in tuberculosis disease: A simulation study. American *Journal of Epidemiology.* 2013;**178(8)**:1281–1288.
94. Strickland SJ, Scott HM, Libal MC, Roussel AJ, Jordan ER. Effects of seasonal climatic conditions on the diagnosis of *Mycobacterium avium* subspecies paratuberculosis in dairy cattle. *Journal of Dairy Science.* 2005;**88(7):**2432–2440.
95. Tedijanto C, Hermans S, Cobelens F, Wood R, Andrews JR. Drivers of seasonal variation in tuberculosis incidence: Insights from a systematic review and mathematical model. *Epidemiology.* 2018;**29(6):**857–866.
96. Thomson RM, Carter R, Tolson C, Coulter C, Huygens F, Hargreaves M. Factors associated with the isolation of Nontuberculous mycobacteria (NTM) from a large municipal water system in Brisbane, Australia. *BMC Microbiology.* 2013;**13(1):**89**.**
97. Thomson RM, Furuya-Kanamori L, Coffey C, Bell SC, Knibbs LD, Lau CL. Influence of climate variables on the rising incidence of nontuberculous mycobacterial (NTM) infections in Queensland, Australia 2001–2016*. Science of the Total Environment.* 2020;**740**:139796.
98. Tian RBD, Niamké S, Tissot-Dupont H, Drancourt M. Detection of *Mycobacterium ulcerans* DNA in the environment, Ivory Coast*. PLoS ONE*. 2016;**11(3):**e0151567.
99. Top R, Boshuizen H, Dekkers A, Korthals Altes H. Similar seasonal peak in clustered and unique extra-pulmonary tuberculosis notifications: Winter crowding hypothesis ruled out? International *Journal of Tuberculosis and Lung Disease.* 2013;**17(11):**1466–1471.
100. Truman RW, Job CK, Hastings RC, Kumaresan JA, Mcdonough CM. Seasonal and spatial trends in the detectability of leprosy in wild armadillos. *Epidemiology and Infection*. 1991;**106(3):**549–560.
101. Uwamahoro D, Beeman A, Sharma VK, Henry MB, Garbern SC, Becker J, et al. Seasonal influence of tuberculosis diagnosis in Rwanda. *Tropical Medicine and Health.* 2021;**49(1):**6.
102. Wah W, Das S, Earnest A, Lim LKY, Chee CBE, Cook AR, et al. Time series analysis of demographic and temporal trends of tuberculosis in Singapore. *BMC Public Health.* 2014;**14(1)**:1–10.
103. Wang H, Tian CW, Wang WM, Luo XM. Time-series analysis of tuberculosis from 2005 to 2017 in China*. Epidemiology and Infection*. 2018;**146(8):**935–939.
104. Wang Y, Xu C, Li Y, Wu W, Gui L, Ren J, et al. An advanced data-driven hybrid model of SARIMA-NNNAR for tuberculosis incidence time series forecasting in Qinghai Province, China*. Infection and Drug Resistance.* 2020;**13**:867–880.
105. Wang Y, Xu C, Ren J, Wu W, Zhao X, Chao L, et al. Secular seasonality and trend forecasting of tuberculosis incidence rate in China using the advanced error-trend-seasonal framework*. Infection and Drug Resistance.* 2020;**13**:733–747.
106. Wang Y, Xu C, Zhang S, Wang Z, Yang L, Zhu Y, et al. Temporal trends analysis of tuberculosis morbidity in mainland China from 1997 to 2025 using a new SARIMA-NARNNX hybrid model. *BMJ Open.* 2019;**9(7):**e028546.
107. Whiley H, Keegan A, Fallowfield H, Bentham R. Detection of Legionella, L. pneumophila and *Mycobacterium avium* *Complex* (MAC) along potable water Distribution Pipelines. International *Journal of Environmental Research and Public Health*. 2014;**11(7):**7393–7405.
108. Willis MD, Winston CA, Heilig CM, Cain KP, Walter ND, Mac Kenzie WR. Seasonality of tuberculosis in the United States, 1993-2008*. Clinical Infectious Diseases.* 2012;**54(11**):1553–1560.
109. Wingfield T, Schumacher SG, Sandhu G, Tovar MA, Zevallos K, Baldwin MR, et al. The seasonality of tuberculosis, sunlight, vitamin D, and household crowding*. Journal of Infectious Diseases.* 2014;**210(5):**774–783.
110. Wubuli A, Li Y, Xue F, Yao X, Upur H, Wushouer Q. Seasonality of active tuberculosis notification from 2005 to 2014 in Xinjiang, China*. PLoS ONE.* 2017;**12(7):**e0180226.
111. Xiao Y, He L, Chen Y, Wang Q, Meng Q, Chang W, et al. The influence of meteorological factors on tuberculosis incidence in Southwest China from 2006 to 2015*. Scientific Reports.* 2018;**8(1):**9072.
112. Xu M, Li Y, Liu B, Chen R, Sheng L, Yan S, et al. Temperature and humidity associated with increases in tuberculosis notifications: a time-series study in Hong Kong*. Epidemiology and Infection.* 2020;**149**:e8.
113. Yang S, Gao Y, Luo W, Liu L, Lei Y, Zhang X. Spatiotemporal distribution of tuberculosis during urbanization in the new urban area of Nanchang city, China, 2010–2018*. International Journal of Infectious Diseases.* 2019;**80**:S120.
114. Yang X, Duan Q, Wang J, Zhang Z, Jiang G. Seasonal variation of newly notified pulmonary tuberculosis cases from 2004 to 2013 in Wuhan, China. *PLoS ONE*. 2014;**9(10)**:e110485.
115. Yang Y, Guo C, Liu L, Zhang T, Liu W. Seasonality impact on the transmission dynamics of tuberculosis. *Computational and Mathematical Methods in Medicine.* 2016;**2016**:8171081.
116. Yu Y, Wu B, Wu C, Wang Q, Hu D, Chen W. Spatial-temporal analysis of tuberculosis in Chongqing, China 2011-2018. *BMC Infectious Diseases.* 2020;**20(531):**1–12.
117. Zhang G, Huang S, Duan Q, Shu W, Hou Y, Zhu S, et al. Application of a hybrid model for predicting the incidence of tuberculosis in Hubei, China. *PLoS ONE*. 2013;**8(11)**:e78216.
118. Zhang YQ, Li XX, Li W Bin, Jiang JG, Zhang GL, Zhuang Y, et al. Analysis and predication of tuberculosis registration rates in Henan Province, China: An exponential smoothing model study. *Infectious Diseases of Poverty.* 2020;**9(1):**1–12.
119. Zhang Y, Ye J, Hou S, Lu X, Yang C, Pi Q, et al. Spatial-temporal analysis of pulmonary tuberculosis in Hubei Province, China, 2011- 2021. *PLoS ONE.* 2023;**18(2)**:e0230877.
120. Zhao D, Zhang H, Cao Q, Wang Z, He S, Zhou M, et al. The research of ARIMA, GM(1,1), and LSTM models for prediction of TB cases in China. *PLoS ONE.* 2022;**17(2)**:e0263545.
121. Zheng Y, Zhang L, Wang L, Rifhat R. Statistical methods for predicting tuberculosis incidence based on data from Guangxi, China. *BMC Infectious Diseases.* 2020;**20(1):**1–8.
122. Zuo Z, Wang M, Cui H, Wang Y, Wu J, Qi J, et al. Spatiotemporal characteristics and the epidemiology of tuberculosis in China from 2004 to 2017 by the nationwide surveillance system. *BMC Public Health.* 2020;**20(1)**:1–12.

# Articles not retrieved

| **Citation** | **Location** | **Mycobacteria Studied** | **Reason** |
| --- | --- | --- | --- |
| Antonio DB et al. (2000). *Prevalence of mycobacterium in wild and captive delta smelt.* | California, USA | *Mycobacterium spp*. | No files available |
| Azhar K, Perwitasari D. (2013). *Physical housing conditions and behaviors with prevalence of pulmonary tuberculosis in provinces of Jakarta, Banten and North Sulawesi.* | Indonesia (Jakarta, Banten, North Sulawesi) | *Mycobacterium tuberculosis* | No files available |
| Bates JH. (1977). *The changing scene in tuberculosis.* | Not specified | *Mycobacterium tuberculosis* | Payed access |
| Coker RJ et al. (2003). *Tuberculosis control in Samara Oblast, Russia: institutional and regulatory environment.* | Samara Oblast, Russia | *Mycobacterium tuberculosis* | Payed access |
| Desikan KV. (1977). *Viability of Mycobacterium leprae outside the human body.* | Not specified | Mycobacterium leprae | Error in publication record |
| Milaat WA. (1994) *Epidemiology of tuberculosis in Jeddah Region, Saudi Arabia* | Jeddah, Saudi Arabia | *Mycobacterium tuberculosis* | No files available |
| George KL et al. (1980). *Epidemiology of infection by nontuberculous mycobacteria. II. Growth and survival in natural waters.* | Eastern United States | Nontuberculous mycobacteria | Payed access |
| Hazra J. (1986). *Tuberculosis in West Bengal.* | West Bengal, India | *Mycobacterium tuberculosis* | No files available |
| Hennessey KA et al. (2000). *Using DNA fingerprinting to detect transmission of Mycobacterium tuberculosis among AIDS patients in two health-care facilities in Puerto Rico.* | Puerto Rico | *Mycobacterium tuberculosis* | No files available |
| Payne A. et al. (2013) *Interactions between wild boars, badgers and cattle in Côte d’Or: Which opportunity for the transmission of Mycobacterium bovis?* | Côte d’Or, France | *Mycobacterium bovis* | No files available |
| Janmeja AK, Mohapatra PR. (2005). *Seasonality of tuberculosis.* | Not specified | *Mycobacterium tuberculosis* | No files available |
| Karandanis D, Shulman JA. (1976). *Recent survey of infectious meningitis in adults: review of laboratory findings in bacterial, tuberculous, and aseptic meningitis.* | Not specified | *Mycobacterium tuberculosis* | No files available |
| Kubálek I et al. (1995). *The spring-fall variations in the prevalence of environmental mycobacteria in drinking water supply system.* | Czech Republic | Environmental mycobacteria | No files available |
| Lefebvre G et al. (1978). *Croissance synchrone en milieu liquide d'espèces liées à Mycobacterium rhodochrous: Nocardia restricta et Jensenia canicruria.* | Not specified | *Nocardia restricta, Jensenia canicruria* | No files available |
| Marston BJ et al. (1995). *Emergence of Buruli ulcer disease in the Daloa region of Cote d'Ivoire.* | Daloa, Côte d'Ivoire | *Mycobacterium ulcerans* | Payed access |
| Masai H. (1972). *[A study on atypical mycobacteria. I. Statistical study on atypical mycobacteria in sputum].* | Japan | Atypical mycobacteria | No files available |
| Omondi PP et al. (1983). *Nontuberculous mycobacterial trends at the Veterans Administration Medical Center in Oklahoma City.* | Oklahoma City, USA | Nontuberculous mycobacteria | No files available |
| Scarlata G et al. (1985). *Isolation of Mycobacteria from drinking water in Palermo.* | Palermo, Italy | Mycobacteria spp. | No files available |
| Nagayama N. *Seasonality of various forms of tuberculosis.* | Not specified | *Mycobacterium tuberculosis* | Payed access |
| Singh S et al. (2016). *Seasonal Variability of Tuberculosis in Sonepat, India.* | Sonepat, India | *Mycobacterium tuberculosis* | No files available |
| Storey MV et al. (2008). *Opportunistic pathogens in drinking and recycled water distribution systems.* | Not specified | Opportunistic mycobacteria | No files available |
| Sutherland I. (1976). *Recent studies in the epidemiology of tuberculosis, based on the risk of being infected with tubercle bacilli.* | Not specified | *Mycobacterium tuberculosis* | No files available |
| Thomas MD et al. (1977). *Tuberculous meningitis (T.B.M.)(a clinical study of 232 cases).* | Not specified | *Mycobacterium tuberculosis* | No files available |
| Cristina BM et al. (2018) *Tuberculosis in wild boar (Sus scrofa) in the western Liguria Region* | Liguria Region, Italy | *Mycobacterium bovis* | No files available |
| Wiwanitkit V. (2010). *MAP in the environment of dairy herds.* | Not specified | *Mycobacterium avium* *subspecies paratuberculosis* (MAP) | No files available |

# Representative articles for quality assessment classification

**Class 0:**
Amofah GK, Adjei-Acquah C, Sagoe-Moses C, Frimpong EH. Epidemiology of buruli ulcer in amansie west district, Ghana. Transactions of the Royal Society of Tropical Medicine and Hygiene. 1993;87(6):644–645.

**Class 1:**

Truman RW, Job CK, Hastings RC, Kumaresan JA, Mcdonough CM. Seasonal and spatial trends in the detectability of leprosy in wild armadillos. Epidemiology and Infection. 1991;106(3):549–560.

**Class 2:**
Ane Anyangwe, Irene & Akenji, T & Beng, Veronique & Titanji, Vincent. (2006). Seasonal variation and prevalence of tuberculosis among health seekers in the South Western Cameroon. East African medical journal. 83. 588-95. 10.4314/eamj.v83i11.9474.

**Class 3:**
Wang Y, Xu C, Zhang S, Wang Z, Yang L, Zhu Y, et al. Temporal trends analysis of tuberculosis morbidity in mainland China from 1997 to 2025 using a new SARIMA-NARNNX hybrid model. BMJ Open. 2019;9(7):e028546.
